# “Bupe by the book”: A study protocol for a pilot randomized controlled trial of library-facilitated telehealth to increase buprenorphine treatment among individuals experiencing homelessness

**DOI:** 10.1186/s13722-025-00599-2

**Published:** 2025-09-17

**Authors:** Lianne A. Urada, Carla Marienfeld, Megan Partch, Richard S. Garfein, Steffanie A. Strathdee, Melanie J. Nicholls, Ashley Weitensteiner, María Luisa Zúñiga, Peter Davidson, Eileen V. Pitpitan

**Affiliations:** 1 University of California San Diego School of Medicine, San Diego, USA; 2 San Diego State University School of Social Work, San Diego, USA; 3 Father Joe’s Villages, Village Health Center, San Diego, USA; 4 Herbert Wertheim School of Public Health and Human Longevity Science, UC San Diego, San Diego, USA

**Keywords:** Homelessness, Unhoused, Unstably housed, Opioid use, Medications for opioid use disorder (MOUD), Buprenorphine, Substance use treatment, Public libraries, Randomized controlled trial

## Abstract

**Background:**

Accessing opioid use disorder (OUD) treatment is difficult for individuals with unstable housing. This population often uses public libraries for computer and internet access, which could provide telehealth access to OUD treatment. Therefore, we developed a novel 12-week library-facilitated telehealth intervention study called “Bupe by the Book” (BBB) that uses library resources to facilitate initiation and retention in OUD treatment with buprenorphine.

**Methods:**

The study is a partnership between the San Diego Public Library and a federally qualified healthcare center attached to a homeless shelter (Father Joe’s Villages (FJV) Village Health Center). We co-designed a pilot randomized controlled trial to assess the feasibility and acceptability of a library-facilitated telehealth intervention in San Diego, California. The intervention is being evaluated for its feasibility and acceptability (library tele-buprenorphine uptake) by obtaining an estimate of the effect of the library telehealth arm of the intervention on buprenorphine treatment outcomes (primary outcome: buprenorphine uptake, i.e., a pharmacy pickup and taking the prescription 1 + times) and adherence (i.e., > 1 buprenorphine positive screens, ideally for 8 + weeks), compared to the control (standard care at the clinic) intervention. Individuals reporting homelessness and OUD (with or without other substance use) are eligible. Forty library patrons will be recruited via flyers, screened for eligibility, and referred to FJV Health Center for in-person initial buprenorphine treatment intake visits. Participants who complete intake are enrolled and randomized to the library-facilitated telehealth condition, which involves using library internet and computer resources for follow-up buprenorphine treatment appointments with the medical provider via library telehealth. In the control condition, participants do not use library telehealth for their buprenorphine care follow up appointments, but rather they go in-person to the clinic or per usual standard care protocols. Feasibility and acceptability of the library telehealth intervention and conduct of the randomized controlled trial are determined by the participants’ use of the library telehealth intervention for buprenorphine treatment, and quantitative and qualitative measures assessing their perceptions of the library telehealth intervention, collected over a 12-week period.

**Discussion:**

The design of this pilot study may support the adoption of library-facilitated telehealth treatment as a feasible and acceptable strategy to engage and retain people experiencing homelessness with OUD in buprenorphine treatment.

**Trial registration:**

This trial was registered prospectively at ClinicalTrials.gov (registration number NCT05872386) on May 24, 2023.

## Background

Recently, overdose deaths in the U.S. have increased by as much as 30% compared to prior years [1]. In San Diego County, California, 814 fentanyl-related deaths occurred in 2022 compared to 462 in 2020 [2]. In the fight to treat opioid use disorder (OUD), efforts to integrate buprenorphine into primary care and other settings has been ongoing for over twenty years as a promising medication that can be prescribed or dispensed by physicians in medical offices [3].

In the U.S., over half a million people experience homelessness [4]. The city of San Diego, California, has the fifth largest unhoused population nationally [5] and a rapidly increasing population with OUD [6]. Nearly 30% of unstably housed persons struggle with mental health and substance use disorders [7]. Many persons with OUD remain houseless and profoundly hard to reach.

Buprenorphine is a highly effective treatment for OUD that has been shown to reduce the risk of opioid overdose [8]. In 2023, overdose deaths decreased nationally by 3% for the first time since 2018 [9]. However, formidable barriers to buprenorphine uptake remain challenging, especially for uptake among persons who are experiencing homelessness [10], accounting for 38.7% of reported overdose deaths across San Diego County in 2022 [2, 11, 12].

Overdose deaths among unhoused individuals in San Diego increased 233% from 2019 to 2023 [2]. Unhoused people are disproportionately impacted by the overdose crisis. Buprenorphine uptake (i.e., a pharmacy pickup and taking the prescription one or more times) and adherence (i.e., more than one buprenorphine positive screen, ideally 8 + weeks) may depend on an individual’s housing status and other issues impacting their ability to participate in treatment.

Typically, patients follow an individualized plan that includes visits for medication management, counseling sessions, and urine drug screenings. However, our preliminary work showed that unstably housed persons with OUD at libraries report barriers to following this plan, including a lack of transportation for in-person visits, a lack of smartphones or computers for telehealth encounters, and a lack of consistent telephone numbers/devices for telephone visits [13, 14].

Other barriers to uptake and adherence, such as stigma, lack of knowledge of medications, high out-of-pocket costs, inconsistent insurance coverage, and ethnic disparities related to medication access, are well-documented [15–23].

Additionally, studies show disadvantages for those with digital literacy barriers [24]. Unhoused individuals are less likely to have reliable interconnectivity [25]. As such, public libraries present themselves as public health opportunities to facilitate telemedicine [25] by providing computers, high-quality internet connection, and private spaces where patrons can conduct confidential telehealth calls. Therefore, one place to find unstably housed persons is the public library.

Nationwide, public libraries face an on-site opioid overdose crisis [27, 28], especially among its unhoused patrons. A randomized survey of five U.S. states showed that 12% reported at least one on-site overdose in the prior year [27]. In another study of Ohio’s 251 public library systems, over 60% of 56 survey respondents indicated awareness of on-site opioid-related incidents (transactions, consumption, or overdose) [28]. Data from our own formative research, according to unstably housed individuals in focus groups in downtown San Diego, revealed as much as “two overdoses a day” occurred just outside the library among those experiencing homelessness and other harms [13, 14, 29–32]. Libraries offer a safe place for many unhoused individuals during the day, and numerous libraries have already taken extraordinary measures to help those with OUD [8, 33]. Some libraries now have on-site social workers, nurses, and peer homeless outreach staff [13, 33–45]. In the downtown San Diego Central Library, library security staff are trained to carry and administer Naloxone to patrons who overdose in the bathrooms and around the immediate vicinity. Because individuals experiencing homelessness and OUD regularly patronize public libraries, a public health opportunity presents itself to offer OUD treatment.

Telehealth interventions for buprenorphine, delivered per national standards, provide patients with buprenorphine prescription access through remote telehealth (audio and video or even audio only) sessions with a prescribing medical provider without having to go to in-person clinic visits as frequently or ever. Research shows that telehealth interventions for buprenorphine effectively overcome barriers to buprenorphine uptake/adherence [10]. After the U.S. Drug Enforcement Administration relaxed requirements for prescribing buprenorphine in March 2020 (during the COVID-19 pandemic), studies began highlighting low-barrier buprenorphine treatment options as more accessible under the national regulatory changes [46–48]. The studies include unhoused persons in San Francisco [49], mobile buprenorphine/case management for homeless veterans [50], and a van-based buprenorphine induction clinic in partnership with a federally qualified health center [51]. A review of telehealth-delivered medications for OUD found that a videoconference intervention led to better substance use treatment retention than an in-person group [47, 48, 51–53]. These studies suggest that telehealth can promote the initiation of buprenorphine.

This protocol describes our “Bupe by the Book” (BBB) study, conducted in collaboration with established local partners, including San Diego Public Libraries and Father Joe’s Villages - Village Health Center (FJV), a federally qualified healthcare center (FQHC). Previously, FJV provided patients with an option for tele-buprenorphine (telehealth for buprenorphine) for those who reported access to a device (e.g., computers, smartphones, tablets, or telephones).

Representing a significant step further, the Bupe by the Book study removes the need for access to a device. Instead, it leverages public library resources (devices and internet) to allow individuals experiencing homelessness to access telehealth care for buprenorphine. BBB is a groundbreaking intervention evaluated using a 12-week, two-arm randomized controlled trial (RCT) design. By testing library-facilitated telehealth, the study informs how unstably housed persons and others can potentially receive buprenorphine care innovatively with minimal clinical/non-clinical requirements (e.g., low-barrier induction offered without a waiting period). 

## Methods

### Objectives and hypotheses

The overall objective of this study is to determine the feasibility and acceptability of utilizing library-facilitated telehealth to provide buprenorphine access to unstably housed people with OUD. Feasibility metrics include recording the number of telehealth visits in the library (with buprenorphine providers) and the proportion of patrons, library, and health providers who say they have preference or feel neutral about library telehealth vs. opposition to it. Acceptability includes interviewing participants throughout and at the end of the study, as well as health providers and library staff, about their acceptability and appropriateness of using library tele-buprenorphine.

Feasibility and acceptability of the library telehealth intervention and conduct of the randomized controlled trial will also be determined by the participants’ use of the library telehealth intervention for buprenorphine treatment, and quantitative and qualitative measures assessing the acceptance of the library telehealth intervention, collected over a 12-week period and at exit. As a benchmark, we anticipate that most participants will be retained in the study and will use the library telehealth intervention as assigned. The libraries and the clinic will also be willing and able to implement the telehealth intervention. Specifically, we will measure feasibility and acceptability in the following ways:

#### Feasibility of the telehealth intervention

1. Proportion of telehealth intervention arm participants who complete at least one telehealth visit appointment at the library.
2. Proportion of scheduled visits with the buprenorphine provider that participants attend via telehealth at the library.
3. We will also document any barriers to telehealth that arise (e.g., internet connection issues).

#### Acceptability of the telehealth intervention

1. Qualitative interviews are conducted during the intervention and via exit interviews asking about the participants’ experiences with telehealth, as well as the library and clinic staff’s experiences with delivering the library telehealth.
2. Survey measures also measure the participants’ experiences with telehealth in general to see if changes occur over the course of the study.

#### Feasibility of RCT to evaluate the current intervention

1. Rate of enrollment (Number of participants per week and per month).
2. Proportion of interested and eligible participants who can successfully complete enrollment in the study.
3. Study retention (proportion of RCT-enrolled participants who complete each follow-up assessment).
4. Proportion of RCT-enrolled participants for whom outcome data are collected.
5. We will track the proportion of participants who indicate willingness to be assigned to either condition (the percent of people who decline to participate because they do not like the randomization aspect of the study should be “none” to determine whether the study is feasible).

As a pilot study that is not statistically powered to assess efficacy, the study will provide effect size estimates of the library-facilitated telehealth BBB intervention on the primary outcomes, buprenorphine uptake and adherence, as measured by (1) presence of buprenorphine in weekly urine drug screenings at the library and at FJV health center, (2) buprenorphine prescription pick-ups, and (3) number and frequency of clinic visits, with secondary outcomes including use of fentanyl (e.g., illicitly manufactured) and other opioids (secondary outcome measured in weekly urine drug screens), and (4) self-reported substance use, mental health, and quality of life measures at 1-,2-,4-,8-, and 12-weeks, compared to a control group.

The control group will undergo the same procedures and receive the same incentives from the study staff in the library in terms of weekly assessments. They will also receive the same care from the clinic, except they will not be instructed to use the library telehealth option for their medical visits like the treatment group until their study participation is over. We hypothesize that patrons assigned to the library-facilitated telehealth group will uptake buprenorphine, pick up their buprenorphine prescriptions from the pharmacies, take buprenorphine after two weeks, and adhere to medical appointments more than the control (in-person clinic visits) group.

As a primary outcome, buprenorphine *uptake* is defined as a pharmacy pickup and taking the prescription one or more times during the study period. Buprenorphine *adherence* is indicated by more than one buprenorphine positive screens taken at the library or at FJV Health Center and ideally continuing to take buprenorphine as prescribed for eight or more weeks. The rationale for the primary outcomes, buprenorphine uptake and adherence, via weekly drug screening, is to try to capture the patterns of buprenorphine uptake and adherence among unhoused populations, a likely more isolated and neglected group when it comes to finding, starting, and adhering to buprenorphine due to being unhoused. Therefore, we measure adherence as a primary outcome with 1 + buprenorphine positive tests, ideally for 8 + weeks of 12 weeks (3 months), rather than the standard 3–12 months adherence.

In the study’s formative stages of intervention development, study staff conducted interviews and focus groups with library patrons (those experiencing homelessness or living in a shelter or treatment program), library staff (i.e., security personnel, librarians, outreach workers), general public/parents, and buprenorphine providers to get reactions to the proposed program and to inform the study design. For example, five focus groups with library patrons were conducted across four libraries in San Diego to assess the potential feasibility and acceptability of the planned intervention. The findings from these interviews and focus groups inform the design of the telehealth intervention at the libraries and the measures that can be used in a randomized controlled trial. For example, we gained insights for recruiting and screening participants for opioids (being discreet in public spaces), enrollment procedures (coordinating with medical providers), and timing of the randomization (after first buprenorphine medical provider visit). Another recommendation made is to have persons with lived experience, especially with substance use recovery and experiences with buprenorphine, on the team. Focus group participants suggested recruitment strategies such as placing flyers at specific library bulletin boards frequently visited by unhoused individuals, which many identify as providing a consistent and reliable source of community information.

Many participants feel that using the library setting for care can facilitate an added layer of discreteness for their clinical care by providing a setting to receive medication for opioid use disorder without fear of going to a clinic. Going in-person to a clinic could result in stigma from being identified as someone receiving substance disorder treatment. Further, several participants view the library as a safe location associated with reduced opportunities for encountering environmental triggers for use that may be present in a clinical setting (e.g. large waiting rooms with other individuals who also use drugs or have other mental health issues, pressure to use drugs from people congregating outside clinics for other services). The participants suggest ways to further keep participation in the study confidential at the library by using telehealth services and conducting study visits in private study rooms.

The focus groups and interviews with medical service providers and library staff helps determine ways to keep the study procedures in line with the usual clinic and library protocols to minimize the need for additional costs and staff time at either place, as well as design a protocol with a great likelihood for implementation after the study. For example, process measures are determined to be conducted by the research staff at intervals commensurate with clinical care for buprenorphine (e.g. in-between and at 1-,2-,4-,8-,12- weeks). The timeframe for determination of buprenorphine uptake outcome measures and follow-up visit schedules are vetted with the medical staff commensurate with the standard of care. The formative data gathering helps inform how we collect our process measures (e.g., meeting regularly with the health center to gather appointment keeping data). The clinic and researchers should conduct regular meetings during the duration of the study to ensure a functional and efficient trial.

### Pilot study design

This multisite study employs a parallel-arm randomized controlled trial design with participants allocated to library-facilitated telehealth visits for buprenorphine treatment (BBB intervention) or standard-of-care clinic-based or telephone visits (control). Participants are interviewed before randomization (baseline) and surveyed again at 1-, 2-, 4-, 8-, and 12-weeks (follow-up) to assess participant characteristics and study outcomes.

The study staff also conducts weekly urine drug screenings in the libraries with participants in both study arms to assess buprenorphine uptake and potential continued use of other drugs including fentanyl and other opioids. However, these additional urine drug screening and weekly follow ups at the library are not part of the usual clinic care or telehealth treatment procedures; instead, the clinic already requires in-person urinary drug screenings. Therefore, the additional library-based drug screenings occur to promote study engagement and retention in the study assessments, and to track outcomes without sole reliance on clinic record data. We also collect medical record and pharmacy pick-up data (via the CURES database described below) as part of the study design. Although these procedures are carefully determined to ensure rigor in the evaluation design, participating in these procedures is not a requirement for the library telehealth intervention itself. Participants can continue to use library telehealth without the need for weekly drug screenings in the library upon completion of the study.

Institutional Review Boards at San Diego State University and the University of California San Diego approved this study.

### Study setting

As part of the randomization plan, the pilot study begins with the San Diego Central Library, a nine-floor public library in downtown San Diego (a high school occupies two floors).

The library has multiple conference rooms and study rooms the public can reserve. The researcher coordinates conference and study room reservations with the library for the three days of the week where the study staff meets with the participants across the two libraries.

Halfway through the targeted recruitment number of 20 participants, we will begin using a second public library site, the Mission Valley Branch Library, to recruit and provide telehealth for this study. The Mission Valley branch allows us to test the feasibility and acceptability of library-facilitated telehealth in a different indoor/outdoor library setting. The branch is smaller and less central than the downtown Central library location. It is also next to public transportation (trolley, a metropolitan commuter train) and the San Diego riverbed, where many persons experiencing homelessness have camped. Like the downtown Central library, drug overdoses have been observed at the Mission Valley branch, making it a second ideal location different enough to demonstrate feasibility and acceptability in different types of library settings.

Before this study, Father Joe’s Villages - Village Health Center, a federally qualified healthcare center (FQHC), already implemented a protocol for free, same-day medication for opioid use treatment. “Same day” means patients can have walk-in appointments (open daily on weekdays) to receive a prescription for buprenorphine from FJV Health Center which they then need to take to a pharmacy to pick-up the medication. Same-day appointment reduces barriers for unhoused patients who may otherwise need to wait for weeks before getting a prescription for buprenorphine at other local medical providers. The availability of this service is one of the reasons we sought to partner with this FQHC for the present research.

However, FJV’s tele-buprenorphine program is not well known, has limited access, and has never been tested with public libraries. The health center is connected to St. Vincent de Paul Village (Father Joe’s Villages) with comprehensive housing and food services in a downtown San Diego location where individuals experiencing homelessness congregate. It is located 0.4 miles from the downtown Central Library and 7.6 miles from the Mission Valley Library.

### Eligibility criteria

Individuals are eligible for this study if they meet the following criteria: (1) reside in San Diego County, California; (2) currently experiencing homelessness by living in a shelter, on the streets, in a car, staying with friends or family, in temporarily rented spaces (e.g., motel), abandoned building, in a tent, or otherwise unstably housed (e.g., evicted during the past month); 3) self-report deliberate use of opioids (urinary drug screening confirmation of opioid use is not required for enrollment, though it is conducted at both the library and clinic sites) within the last three months or as determined eligible for buprenorphine by the medical provider at the clinic (i.e., individuals are not eligible if they only report unintentional use of opioids, for example when other drugs used are found to be adulterated with fentanyl); 4) not currently receiving medications for opioid use disorder (such as methadone, buprenorphine, naltrexone) from another medical provider, and 5) express the desire to quit or reduce opioid use by taking buprenorphine.

### Screening and enrollment

Participants are recruited via flyers posted at reference desks and kiosks/bulletin boards at the San Diego Central and Mission Valley libraries. Non-library personnel are not allowed to approach patrons inside the library to respect their privacy. Therefore, in-person encounters within the libraries occur only when a patron approaches the research staff. The research staff sit in reserved library conference rooms. At the Mission Valley library, tables with study information are set up outside and inside the entrance to advertise the study. At the downtown Central Library, a librarian can occasionally introduce the research study over the public announcement system to refer patrons. Social work student interns formally working at the library can also refer patrons who see them for other needs. In addition, patrons may approach the library front desk or security to inquire about the study after seeing a flier or if they hear about the study from others.

Trained research staff also prescreen interested individuals for enrollment in the study during outreach outside the perimeters of the library. Recruitment around the perimeter of the libraries is sometimes necessary to spread the word about the research study. Further, because the Mission Valley library’s location is not as central in a commercial district, study staff can recruit participants in the immediate areas of the two metropolitan transit system trolley stops closest to the Mission Valley library and along trails above the San Diego riverbed where many unhoused individuals camp.

Unstably housed individuals who express an interest in obtaining buprenorphine to treat their current opioid use are invited to a reserved public library room for screening. In-person screenings are then conducted by research staff using a computer-assisted questionnaire to assess eligibility. The researchers obtain written informed consent from the participants to access their FJV Health Center medical record data about their medical appointment adherence for opioid use disorder and drug screenings on-site. Participants also consent to researchers searching CURES (Controlled Substance Utilization Review and Evaluation System), a state-level database of dispensed controlled substance prescriptions, allowing researchers to confirm whether participants picked up their controlled medications from the pharmacy. Participants indicate their preferred language for communication (English or Spanish). Researchers fluent in the selected language then review the corresponding consent form, also in their preferred language, with the participants.

For both the library-facilitated telehealth and control arms of the study, study enrollment is initiated by the study staff; enrollment is completed when the participant attends their first medical buprenorphine appointment in-person at the FJV Health Center and a medical provider confirms their eligibility for buprenorphine. Participants travel by foot from the downtown library (10-minute walk) or by trolley/bus from the Mission Valley Library to FJV Health Center. Participants receive transportation passes throughout the study. FJV prefers patrons attend the first clinic visit in-person for closer screening of buprenorphine eligibility. The study will track the number of patrons who initiate enrollment but do not make it to FJV to complete their enrollment.

### Randomization and intake

After confirmation of buprenorphine eligibility from a FJV Health Center buprenorphine provider, study staff complete study enrollment and randomize participants into one of two study arms. Both library telehealth and control group participants then complete interviewer-administered surveys and follow-up interviews over the 12 weeks of the study conducted at the libraries. Study staff contact all patrons in both study arms, including those who missed appointments or do not return to participate in the research study at the libraries, to help reconnect them to the research study.

### Study materials and incentives

For each baseline and follow-up visit for library-facilitated telehealth and control arms, all participants completing assessments with the library researcher staff receive $20 cash (gift card optional), transportation day passes per library visit with the researcher, and snacks/drinks.

### Intervention condition

In the intervention group, participants are scheduled for regular appointments at the FJV Health Center, which they attend via telehealth at the library. However, they can also access telehealth with FJV Health Center at unscheduled times. Unscheduled drop-in appointments is a standard available option for FJV Health Center patients and can be extended to library telehealth. The ability of unhoused participants to keep their regular buprenorphine appointments, whether in-person or by library telehealth, is a process measure for the study.

In some situations, participants can be interviewed outside of the library, if a room is not available in the library or the participant has too many belongings to bring inside the library.

Efforts are made to keep appointments private in locations not within hearing distance of others. The libraries have a computer tablet on a telehealth floor stand in a private study room for this study. The libraries make the computer tablet available on a heavy platform (to prevent theft) that can be placed in a library study room. The tablet is an alternative that removes barriers for patrons without ID cards who would otherwise need an ID card to check out library laptops.

FJV medical providers follow standard of care practice (whether in-person visit or over telehealth) to provide medication adherence counseling, medication education (e.g., how to take buprenorphine), information about what to expect, appointments, and treatment goals (e.g., relapse prevention, health/mental health referrals). Researchers help make follow-up appointments with the FJV Health Center for both the control and telehealth library groups, and research or library staff can also assist patrons with logging on to their telehealth appointments.

As part of usual care for participants in both study arms, participants are still expected to attend the FJV Health Center in-person when asked to do a follow-up urinary drug assessment at the clinic, despite participating in the drug screenings weekly at the library. Participants in the library-facilitated telehealth arm are not forced to use the telehealth option solely; they can still attend in-person medical visits (provide clinic urine samples), Alcohol and Drug (AOD) counselor visits, and support groups at the clinic. Therefore, the continued in-person visits to the clinic and uptake of library telehealth is another process measure. As described below, the control group is only offered library-facilitated telehealth once they complete their 12-week study participation.

### Control condition

Like the library-facilitated telehealth condition, participants in the control condition receive the same medical treatment in phases based on an individual case basis, case management referrals by research staff, research incentives, and face-to-face follow-ups by the researchers at the library. The only difference between the study arms is that control participants are not offered library-facilitated video sessions during the 12-week participation in the study.

However, after completing the 12 weeks in the study, control participants have the option of library-facilitated telehealth.

### Outcome ascertainment and post-intervention assessments

Intervention outcomes determine the feasibility, acceptability, and retention in the library telehealth intervention at baseline, 1-,2-,4-,8-, and 12-weeks on (1) weekly urine drug screenings (for buprenorphine uptake and other drugs); (2) buprenorphine prescription pick-ups; (3) clinic visits; and (4) self-reported measures (e.g., substance use, mental health, quality of life). (see Table 1).

**Table 1:** BBB intervention targets, strategies, and specific activities

**Intervention targets**	**Intervention strategies**	**Examples of specific activities**	**Data Collection Format**
Feasibility and acceptability of library telehealth for buprenorphine uptake (pharmacy pickup, taking prescription one or more times) and adherence (more than one buprenorphine positive screen or taking buprenorphine for 8 + weeks)	Participants use library telehealth. Data are used to assess participant satisfaction (in exit interviews) and identify areas for improvement.	All participants in the library-facilitated telehealth and control groups complete a post-treatment satisfaction survey regardless of whether they sustained in treatment. At the end of the participants’ 12-week participation in the study, research staff interview participants about their experience with library telehealth and the acceptability of BBB study participation. The investigators survey medical providers and library staff involved in the study about their experiences with the study protocol.	Surveys and interviews, delivered by a researcher not involved in their care. Satisfaction measures are embedded in the follow up exit survey and interview using specific items generated by the study about the telehealth and study experiences.
Awareness of buprenorphine availability	Basic buprenorphine education, increase in knowledge of service providers.	General information on buprenorphine availability at Father Joe’s Villages and in the San Diego region.	Personalized discussion, resource list delivered by researchers and library social work interns.
Apprehension toward buprenorphine*	Basic buprenorphine education, increase knowledge of health.	General background on buprenorphine treatment (e.g., targeting of opioids). Facts about buprenorphine. Buprenorphine treatment information.	Brief educational information sheets delivered by researchers/library social work interns and FJV Health Center staff.
Limited MOUD treatment skills	Problem-solving	Identifying challenges relevant to individuals in continuing through treatment (e.g., personal identification for pharmacy, contact information for reminders).	Personalized discussion to address barriers delivered by researchers/library social work interns and FJV Health Center staff.
Limited telehealth behavioral skills	Modeling	Modeling process of logging in to the telehealth system. Education on what medical professionals to expect and when (e.g., nurse followed by the doctor).	Demonstration delivered by researchers/library social work interns.

*Buprenorphine Apprehension. Though individuals want to try buprenorphine to help them treat their opioid use, some fear of possible significant side effects, such as precipitated withdrawals. Others perceive buprenorphine would not work for them or they do not know what it is

### Data collection

#### Baseline and longitudinal assessments/measures

Following a FJV Health Center physician’s buprenorphine prescription, research staff track the participants’ buprenorphine medication uptake (pharmacy pickup, taking prescription one or more times during the first two weeks in the study), adherence (more than one buprenorphine positive screen), and medical appointment keeping through urinary drug screenings, interviews conducted at public libraries, and medical record data. All participants are invited to return to the library weekly for urinary drug screenings and to complete follow-up survey assessments at weeks 1, 2, 4, 8, and 12. Baseline and follow-up assessments are conducted in reserved library study rooms or conference rooms.

The 60-minute baseline survey captures the following domains: sociodemographic, behavioral, social supports, telehealth utilization and comfort, library usage, substance use behaviors, overdose experiences, and treatment history, along with the biomarker and medical record data collection (see Table 2). The content of the follow-up surveys is the same as the baseline surveys except that participant history and demographics are removed. The follow-up survey is shorter (30 min), retaining questions surrounding buprenorphine uptake, healthcare utilization, and barriers and facilitators to use. Follow-up questions also ask participants to recall experiences since their last visit with the research team in the library, particularly to determine the feasibility and acceptability of the library telehealth intervention; ease and understanding of being in the randomized controlled trial; experiences following up with the medical provider whether in-person or by telehealth; and their experiences with picking up and taking the buprenorphine medication.

Urinary drug assessments at the library are not part of the intervention, but rather part of the research assessment procedures and to better track participants over time (a strategy for reducing loss to follow up). Urinary drug assessments for buprenorphine are still required in-person at the clinic as the routine medical procedures for both arms of the study, a standard procedure even during the COVID-19 pandemic at FJV Health Center. The urinary strips at the library are dipsticks used to assess for buprenorphine, fentanyl, and other drugs to assess for the primary outcome of the study (buprenorphine uptake and adherence) and secondary outcome (reduction of fentanyl and other drugs). The participants use library bathroom facilities to collect their weekly self-administered urinary drug screening tests. Patrons take the urinary drug test strips (for buprenorphine, fentanyl, and other drugs) and a plastic cup to the library bathrooms and return the test strips in a clear hazard bag for the study staff to record in a de-identified log. After each visit with research staff, participants receive 20 dollars cash and a Metro Transit System pass(es) as reimbursement for their time and travel needs to and from their clinic or library appointments. As noted above, these urinary drug assessments in the library and incentives/compensation are not required or offered for the telehealth intervention in the library after the study ends. Only the library telehealth would still be available.

We anticipate that many participants do not have a cellular phone or reliable phone access as phones are often lost, stolen, or run out of minutes due to the individual’s economic vulnerabilities, which exposes them to theft from other individuals, lack of income to pay the bill, and “sweeps” or forcible displacement from law enforcement. For follow-ups, the research staff obtain alternate phone numbers and emails of other individuals who can locate the participant. However, participants will be provided with information on the days and times the research staff are at the library and can simply show up at those designated times (two or three days per week). Researchers also keep library staff and security informed of the study location within the library and times of researcher availability to facilitate follow-up meetings with participants.

**Table 2:** Measures

Topic/Variable	Covariate Measure	Survey instrument/measure
**Feasibility and Acceptability of the Intervention and RCT study design**	Example: You were randomly assigned to either telehealth in the library with your FJV prescribing doctor or in-person follow-ups with your FJV prescribing doctor. Which would have worked better for you and for what reasons?	Study generated (open and close ended questions) *Feasibility of the telehealth intervention*: Participant completes 1 + appointment with the buprenorphine provider via telehealth at the library. Document barriers to telehealth that arise (e.g., internet connection issues) *Acceptability of the telehealth intervention*: Qualitative interviews during the intervention and via exit interviews asking about the participants’ experiences with telehealth, as well as the library and clinic staff’s experiences with delivering the library telehealth *Feasibility of RCT to evaluate the current intervention*: Rate of enrollment (Number of participants per week and per month), Proportion of interested and eligible participants who can successfully complete enrollment in the study, Study retention (proportion of RCT-enrolled participants who complete each follow-up assessment), Proportion of RCT-enrolled participants for whom outcome data are collected.
**Primary Outcomes**		
Buprenorphine uptake and adherence	Biomarkers: urinary drug assessments at the library and clinic. Pharmacy pickups of buprenorphine Self report: “How often do you take it? What is the dosage? Are you taking it correctly (as prescribed)? If not, why?” “Did you run out of your prescription before getting a refill?” “Have you stopped taking buprenorphine?”	Study generated Uptake: the proportion of people who pick up from the pharmacy and take buprenorphine 1 or more times at initiation, i.e., during the first 2 weeks, confirmed by biomarkers (urinary drug screens at the library and clinic, pharmacy pickup data from the state’s CURES database) Adherence = more than one buprenorphine positive screen taken at the library or at the FJV Health Center, ideally for 8 + weeks.
**Secondary outcomes**		
Medical appointment-keeping	Medical record data and self-reported survey data	Study generated
Substance Use (reduction or abstinence for 7 + days)	NIDA-Modified ASSIST V2.0 [54] Biomarkers: urinary drug assessments of fentanyl, opioids, and other drugs (i, e., methamphetamines, cocaine, MDMA, other opioids)	http://www.sbirtoregon.org/wpcontent/uploads/Modified-ASSIST-English-pdf.pdf https://nida.nih.gov/sites/default/files/pdf/nmassist.pdf
Alcohol Use	AUDIT-C [55]	https://cde.nlm.nih.gov/formView?tinyId=myWNfJaZwe
Motivation (e.g., to stop using opioids, to take buprenorphine)	Readiness Ruler [56]	https://case.edu/socialwork/centerforebp/resources/readiness-ruler
Depression and Anxiety	PHQ-4 [57]	https://www.oregonpainguidance.org/app/content/uploads/2016/05/PHQ-4.pdf
Childhood Trauma Experiences	ACES [58]	https://www.acesaware.org/wp-content/uploads/2022/07/ACE-Questionnaire-for-Adults-Identified-English-rev.7.26.22.pdf
Social Support	MOS Social Support Survey [58], [59], [60]	https://pubmed.ncbi.nlm.nih.gov/2035047/
Experience with Technology	Adapted from Garfein, et al. VDOT study [60]	Garfein, et al. (2018). Tuberculosis Treatment Monitoring by Video Directly Observed Therapy in 5 Health Districts, California, USA. *Emerging infectious diseases*, *24*(10), 1806–1815. 10.3201/eid2410.180459
Library Usage	Questions around individuals use of the library (e.g., time spent visiting, activities at library, frequency of visits)	Study generated
OUD Treatment Histories	Questions eliciting information on prior pharmacological or psychological treatment of opioid use disorder (e.g., methadone, counseling)	Study generated

At the end of the participants’ 12-week participation in the study, research staff interview participants about their experience with library telehealth and the acceptability of BBB study participation. To avoid potentially biased responses, interviews are conducted with study staff not involved in the person’s care. All participants in the library-facilitated telehealth and control groups complete a post-treatment satisfaction survey regardless of whether or not they sustain in treatment. Data are used to assess participant satisfaction and identify areas for improvement.

The investigators will also interview the medical providers and library staff involved in the study to get feedback about their experiences with the study protocol.

#### Data tracking and management

For tracking, the researcher assigns each participant a unique identifier number, and the study staff records and maintains each session’s date, time, and location. The researchers use the electronic tracking sheet to record drug screening results (buprenorphine, fentanyl, and other substances) collected on-site at the libraries for the research study. They also record participant receipt of incentives, study staff initials, and the type of assessment they administer (baseline or follow-up). Notes with de-identified data are also recorded in the spreadsheet.

Additionally, participant medical appointment attendance, clinic urinary drug screening outcomes, and CURES database-derived pharmacy pickup data are recorded on a separate HIPAA-compliant database to be shared only with the clinic staff on the study and selected research staff.

#### Sample size and planned data analyses

Our target sample size for the trial is 40 participants, although we allow for a maximum of 60 participants to account for attrition. The sample size will provide adequate precision for effect size estimates, though not statistically powered to assess efficacy on primary outcomes.

Buprenorphine uptake (the proportion who pick up and take buprenorphine at initiation, confirmed by CURES database pharmacy pickup records and urinary drug screenings) and adherence (taking buprenorphine > 1 time, ideally for 8 + weeks), medical appointment adherence (attended medical appointments for buprenorphine two or more times as confirmed by clinic medical record data) will be divided by the number prescribed and scheduled, respectively.

Adherence estimates from this study will also provide the basis for power calculations in the design of future efficacy trials. Quantitative data analysis will consist of univariate statistics to describe the entire study sample and by study arm, the prevalence of individual responses, and the prevalence of treatment uptake and adherence. We will conduct an intent-to-treat (i.e., per-randomization) analysis following CONSORT guidelines to explore potential differences between study arms on the outcomes. Specifically, we will use regression models testing each outcome separately (using the appropriate test depending on the outcome distribution, e.g., logistic regression with binary outcomes). These models will treat the intervention condition (library-facilitated telehealth intervention vs. treatment-as-usual control) as the exposure in unadjusted models.

#### Timeline and dissemination plans

Results from the BBB study will be disseminated widely to organizational, clinic, and library partners, community networks, and nationally at substance use and library information science conferences in consultation with our community partners. Summary findings for community members (non-scientists) will be written following best practices for providing medical information to the public [61]. We will share results with our research partners and stakeholders (e.g., County) via open-access peer-reviewed publications, reports, news media outlets, and conference and community presentations. We aim to eliminate barriers to future Phase III efficacy trials by including residents, patients, providers, and libraries’ input before the study’s pilot. We intend to disseminate this information to these groups through such outlets.

**Fig. 1:**
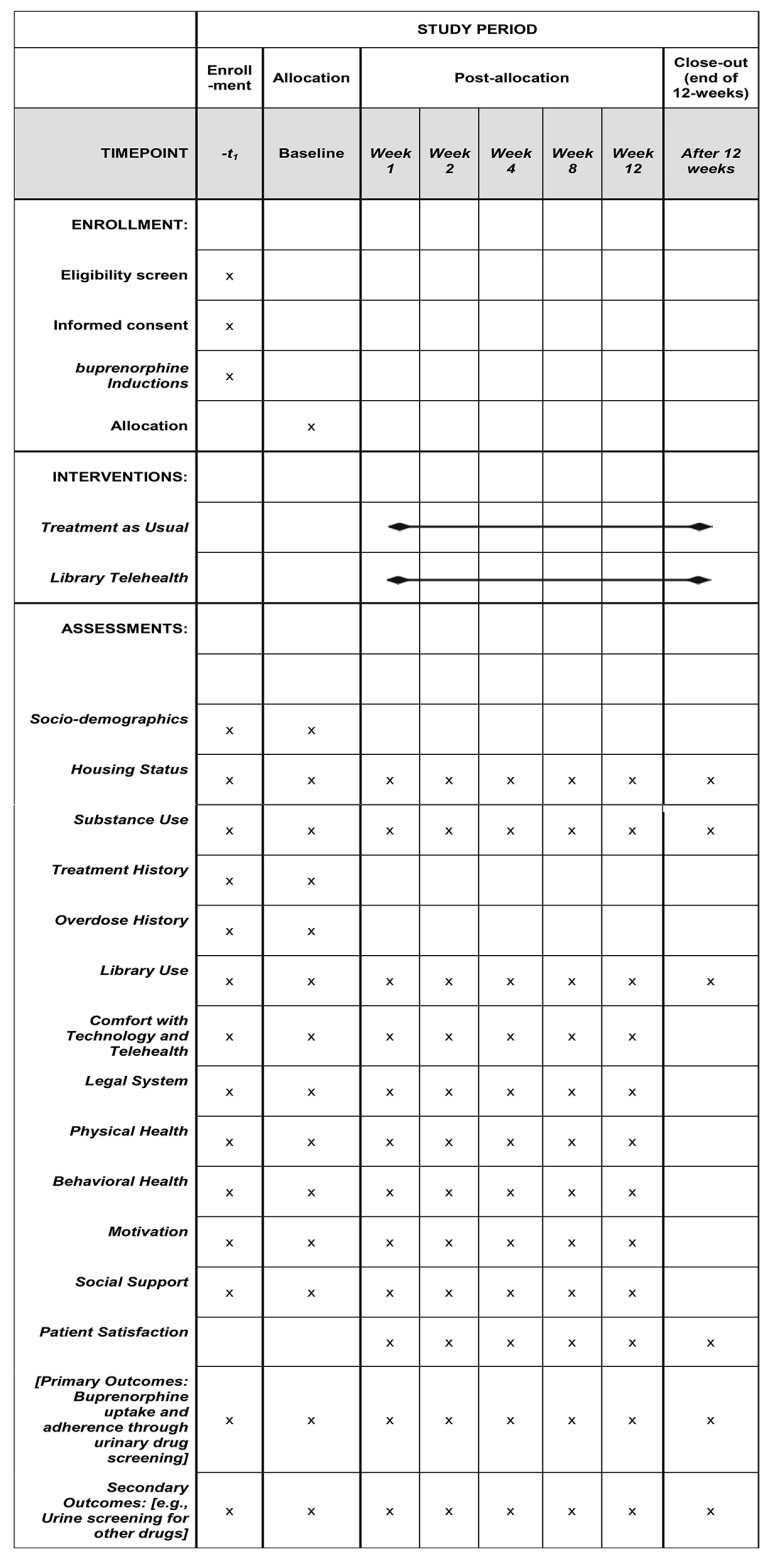
Schedule of enrollment, interventions, and assessments*

Effect size estimates may also inform subsequent intervention research, including a fully powered efficacy trial, with this population and setting (see Fig. 1).

## Discussion

This community-based randomized controlled trial pilot aims to improve buprenorphine uptake and adherence among persons with opioid disorders experiencing homelessness in San Diego County at a public space where they are at already- the public library. Co-created with two San Diego Public Libraries and Father Joe’s Villages’ federally qualified healthcare center, the pilot study attempts to demonstrate the library telehealth’s feasibility and acceptability among the unhoused library patrons with opioid use disorder, libraries, and the clinic staff.

### Conceptualizing each aspect of the intervention

In partnership with the researchers, the libraries adapt FJV Health Center’s free tele-buprenorphine treatment to their space using available resources to ensure its sustainability. Meeting rooms in the libraries, internet and library electronic device access, and a phone line in one library are available without additional costs or burden to the library beyond what they would ordinarily make available to the public. The two libraries are carefully selected after conducting interviews and focus groups at four libraries; two are selected for their on-site opioid overdose crises occurring and the easy proximal access to the clinic by foot or public transportation from their locations.

Both the libraries and clinic want the library telehealth intervention to continue without additional or continued cost once the research study ends. FJV Health Center and the researchers discuss procedures to minimize impact on the clinic staff, being intentional about following protocols already set in place by the clinic. The researchers’ study procedures for the sole purpose of data collection and tracking are not intentionally designed to be part of the sustainable intervention; only the library tele-buprenorphine via FJV is the intervention under study.

However, the research staff’s role at the library is similar to a social worker/case manager’s role that exists at some other U.S. libraries. In this case, the researchers help the participant navigate the steps of getting to the first medical appointment in-person to be enrolled in the study. In addition, they follow up with the participants and talk with them when conducting the interviews/surveys, which participants may perceive as an intervention in itself by virtue of interaction at a time in their lives when they have few others with whom to speak and be heard.

Requiring an in-person clinic visit prior to enrollment in the study was informed by shifting public policy. Federal legislation expanded physicians’ ability to initiate and maintain pharmacological treatment of opioid use disorder via telehealth (i.e., without an in-person medical evaluation) during the COVID-19 emergency “stay-at-home” public health policies. With mounting evidence supporting the use of telehealth and its benefits in reducing barriers to buprenorphine access, an extension to use telehealth instead of in-person clinic visitation was granted. Then, it was codified into permanent law [52].

As planning for our study continued, the federal regulation requiring at least one in-person visit was under debate. Because the clinic medical staff preferred to see the patients in-person before providing a prescription for buprenorphine for the first time, we will retain this as part of the study protocol even if laws shift to allow all virtual telehealth visits to be held without an in-person visit. We also stay with one clinic in order to not introduce other variables.

Though higher attrition may occur from screening to enrollment because of the in-person first clinic visit usual care and requirement, the full-scale future trial would adhere to this requirement because of the clinic’s standard practice to see a patient in-person for the first visit. The libraries’ proximity (walking distance or near to a trolley/public transportation stop) may introduce selection issues, excluding those without transportation passes or those unwilling to go to the downtown setting of the clinic. However, all participants in both control and intervention groups receive bus passes from the study. Both groups receive the same treatment by the research staff, library, and clinic so that we can attribute outcomes to the library telehealth intervention through a rigorous trial.

Enrolling participants only after they attend an in-person buprenorphine intake visit with a medical provider at the affiliated clinic may have implications for interpreting the feasibility data and generalizability. However, it is not possible to evaluate outcomes in this trial without this design decision if the goal is to have rigor through an RCT. This intentional design decision avoids enrolling participants who continue in the study for 12 weeks without ever seeing a medical provider as it would not allow us to detect the impact of the telehealth library intervention vs. usual in-person care.

### Impact of the intervention on the library

A challenge can be selecting an appropriate library with suitable infrastructure to allow for the study activities. Of four libraries we partnered with before conducting the RCT, we considered one in a mainly Latinx neighborhood of San Diego. However, we decide to cultivate the relationship with the community for future consideration instead because (1) the community surrounding the library is improving the area, and some focus group participants think the project may attract patrons who are not yet using the library in high numbers, (2) key informant community members say we would need to proceed carefully with participants who are not from the community who come to the library for the research study because they could become targets of territorial gangs. Therefore, another library could be a more practical choice, such as the ones we select for their high number of previous overdoses. For example, we choose to use one for its location above a riverbed, where many unhoused individuals stay, and its proximity to the public transportation trolley line.

### Strengths and limitations of the study design

Having drop-in clinic appointments available in both the telehealth library and in-person clinic control conditions is both a strength and limitation of the study. Drop-in appointments are a low barrier feature of the federally qualified healthcare center’s protocol that works well for unhoused patients. However, if a clinic setting does not allow drop-in clinic appointments, the implications are that a social worker in the library or health navigator will be even more necessary in real-world clinical practice to ensure patrons make and navigate their appointments on time. Usually, an appointment is made, but the clinic allows patients to see the medical provider within the block of time (i.e., morning or afternoon). If they do not show up at their appointment time, they can still see the medical provider within that morning or afternoon shift. Or, they can drop-in any other day on a first-come, first-serve basis. Generalizability is further limited by the requirement to have in-person visits for the first visit, though even during the COVID-19 pandemic when visits were mostly virtual, visits for in-person urine drug screenings were still required by FJV and other health centers.

Furthermore, if the rationale for the library-based intervention is to increase access to treatment, the frequency of urine tests and research assessments in this pilot study is greater than what is typically required in contemporary office-based buprenorphine treatment in many areasof the country. For this study, we want to have frequent follow-ups to ensure a higher likelihood of follow up to retain participants in the study protocols (surveys, interviews). However, an unintended consequence might be that participants will retain on buprenorphine more just by being in frequent contact with study staff. However, because we follow up weekly with all participants, regardless of the treatment vs. control condition, it does not impact our comparisons between conditions. An implication might be that having staff such as library social workers interacting with participants is important in carrying out such an intervention.

In addition, the twenty-dollar weekly incentive for the participants’ time doing follow-up assessments, although a moderate, non-coercive amount, may motivate their initial interest and retention in the study. However, the incentive should not have an effect on the uptake and retention of buprenorphine because participants do not receive any additional incentive for taking buprenorphine medication. The participants only receive incentives, including snacks/drinks and transportation passes, to compensate them for their time and travel to the study sites. Again, both the control and intervention group receive the same incentives. At the same time, incentives may be the key to engaging a hard-to-reach unhoused population into any kind of assistance in the first place.

Regarding treatment retention, pilot data would need to be interpreted with caution because social workers or library staff may not be available to assist the patrons with telehealth in the library afterwards or at other sites. However, overall, the study design attempts to enable library-facilitated telehealth to be sustained with minimal extra work for libraries and clinics. Some patrons might be able to do so more independently than others which would be interesting to study in itself. When planning for an efficacy trial, understanding determinants of successful implementation, including sustainability (and costs) of the treatment model will be taken into consideration for the full-scale trial.

## Conclusion

By testing library telehealth for buprenorphine uptake and adherence, this study may demonstrate how persons experiencing homelessness can receive buprenorphine care innovatively with minimal clinical/non-clinical requirements (e.g., low-barrier induction) in community-based settings. By meeting unhoused people in locations where they already are and by providing care regardless of medical insurance coverage, this protocol attempts to increase access to medications for opioid use disorder to unhoused people, extending beyond overdose prevention-only measures at public libraries.

## Data Availability

No datasets were generated or analysed during the current study.

## Abbreviations

BBB: Bupe by the Book
COVID-19: Coronavirus Disease
CURES: Controlled Substance Utilization Review and Evaluation System
FJV: Father Joe’s Villages Village Health Center
MOUD: Medications for opioid use disorder
OUD: Opioid use disorder
RCT: Randomized controlled trial
